# A novel algorithm for alignment of multiple PPI networks based on simulated annealing

**DOI:** 10.1186/s12864-019-6302-0

**Published:** 2019-12-27

**Authors:** Jialu Hu, Junhao He, Jing Li, Yiqun Gao, Yan Zheng, Xuequn Shang

**Affiliations:** 1 School of Computer Science, Northwestern Polytechnical University, West Youyi Road 127, Xi’an, 710072 China; 2 Centre of Multidisciplinary Convergence Computing, School of Computer Science, Northwestern Polytechnical University, 1 Dong Xiang Road, Xi’an, 710129 China; 3 Ming De College, Northwestern Polytechnical University, Feng He Campus, Xi’an, 710124 China

**Keywords:** Network alignment, PPI networks, Simulated annealing, Optimization, Functional conserved proteins

## Abstract

Proteins play essential roles in almost all life processes. The prediction of protein function is of significance for the understanding of molecular function and evolution. Network alignment provides a fast and effective framework to automatically identify functionally conserved proteins in a systematic way. However, due to the fast growing genomic data, interactions and annotation data, there is an increasing demand for more accurate and efficient tools to deal with multiple PPI networks. Here, we present a novel global alignment algorithm NetCoffee2 based on graph feature vectors to discover functionally conserved proteins and predict function for unknown proteins. To test the algorithm performance, NetCoffee2 and three other notable algorithms were applied on eight real biological datasets. Functional analyses were performed to evaluate the biological quality of these alignments. Results show that NetCoffee2 is superior to existing algorithms IsoRankN, NetCoffee and multiMAGNA++ in terms of both coverage and consistency. The binary and source code are freely available under the GNU GPL v3 license at https://github.com/screamer/NetCoffee2.

## Introduction

Protein function is a fundamental problem that attracts many researchers in the fields of both molecular function and evolution. Proteins were involved in almost all life processes and pathways. Although many researchers have put a great of efforts to develop public protein annotation databases, such as Uniprot [1], NCBI protein, RCSB PDB [2] and HPRD [3], the task of protein characterization is far to be completed. Thanks to the development of next-generation sequencing [4], computational methods become a major strength for discovering the molecular function and phylogenetic [5–17].

Global network alignment provides an effective computational framework to systematically identify functionally conserved proteins from a global node map between two or more protein-protein interaction (PPI) networks [18–20]. These alignments of two networks are called pairwise network alignment [21, 22]. These of more than two are termed as multiple network alignment [23–25]. The node map of a network alignment is actually a set of matchsets, which consists of a group of nodes (proteins) from PPI networks [24]. There are two types of node maps: one-to-one and multiple-to-multiple. In a one-to-one node map, one node can match to at most one node in another network [26]. In a multiple-to-multiple map, each matchset can have more than one node of a network. With a global network alignment, one can easily predict function of unknown proteins by using “transferring annotation”.

IsoRank was the first algorithm proposed to solve global network alignment, which takes advantage of a method analogous to Google’s PageRank method [27]. An updated version IsoRankN was proposed to perform multiple network alignment based on spectral clustering on the induced graph of pairwise alignment score [28]. Intuitively guided by T-Coffee [29], a fast and accurate program NetCoffee [30] was developed to search for a global alignment by using a triplet approach. However, it cannot work on pairwise network alignment. There are four major steps in the program: 1) the construction of PPI networks and bipartite graphs; 2) the weight assignment based on a triplet approach; 3) the selection of candidate match edges; 4) optimization with simulated annealing. To improve the edge conservation, a genetic algorithm MAGNA was proposed, which mimics the evolutionary process [26]. It starts with an initial population of members. Each member is an alignment. Two members can produce a new member with a crossover function. A fitness function was designed to evaluate the quality of alignments in each generation. MAGNA++ speeds up the MAGNA algorithm by parallelizing it to automatically use all available resources [31]. A more advanced version multiMAGNA++ was applied to find alignment for multiple PPI networks [32]. However, there still exists a gap between network alignment and the prediction of unknown protein function in a systematical level, due to the large amount of molecular interactions and the limitation of computational resources.

Here, we present a novel network alignment algorithm NetCoffee2 based on graph feature vectors to identify functionally conserved proteins. A target scoring function was used to evaluate the quality of network alignment, which integrates both topology and sequence information. Unlike NetCoffee, NetCoffee2 can perform tasks of both pairwise and multiple network alignments. Furthermore, it outperforms existing alignment tools in both coverage and consistency. It includes three major steps: 1) calculation of sequence similarities for pairs of nodes; 2) calculation of topological similarities; 3) maximizing a target function using simulated annealing.

## Definition and notation

Network alignment is a problem to search for a global node mapping between two or more networks. Suppose there is a set of PPI networks {*G*_1_,*G*_2_,...,*G*_*k*_},*k*≥2, each network can be modeled as a graph *G*_*i*_={*V*_*i*_,*E*_*i*_}, where *V*_*i*_ and *E*_*i*_ represents proteins and interactions appearing in networks. A matchset consists of a subset of proteins from $\bigcup _{i=k}^{k} V_{i}$. A global network alignment is to find a set of mutually disjoint matchsets from a set of PPI networks. Note that, each protein can only appear in one matchset in a global alignment solution. Each matchset represents a functionally conserved group of proteins. Pairwise network alignment aims to find an alignment for two PPI networks, whereas multiple network alignment aims to find an alignment for more than two PPI networks. Unlike the previous algorithm NetCoffee, our updated version NetCoffee2 can be applied to search for both pairwise network alignment and multiple network alignments.

## Method

### An integrated model

Sequence information is one of important factors in charactering biological function of genes, RNA and proteins[33]. For example, proteins of a typical family not only share common sequence regions, but also play similar roles in biological processes, molecular function and cellular component. As only a small fraction of a protein sequence is in the functional region, a sequence-based similarity measure is insufficient for the annotation of protein function [34]. PPI network topology can provide complementary information for the prediction of protein function. As used in many other network aligners such as IsoRank, Fuse [35] and Magna, both topology and sequence information are integrated in one similarity measure to search for functionally conserved proteins across species. There are two basic assumptions underlying this methodology: 1) a sequence similarity implies functional conservation; 2) functions are encoded in topology structure of PPI networks.

### Sequence-based similarity

Intuitively guided by an assumption that structures determine functions, most of existing network aligners use both amino acid seqeuences and network topology to predict protein functions. Here, we performed an all-against-all sequence comparison using BLASTP [36] on all protein sequences. These protein pairs with significant conserved regions are taken into consideration for further filtrations. Note that e-value is an input parameter to control the coverage of network alignment. Let *Ω* denote the candidates of homology proteins. Given a protein pair u and v, the sequence similarity s (u,v) can be calculated in the following formula, s _*h*_(u, v)=$\frac {\varepsilon (u, v)-\varepsilon _{min}(u, v)}{\bigtriangleup \varepsilon }$. Here, *ε*(u,v) can be log(evalue) or bitscore of the protein pair u and v, and △*ε* is the largest difference between any two pairs of homolog in *Ω*,△*ε*= *ε*_*max*_(*u*,*v*)−*ε*_*min*_(*u*,*v*), which servers as a normalization factor. The most similar one is 1, the least 0.

### Topology-based similarity

As protein functions are also encoded in the topology of PPI networks, topological structure can guide us to find functionally conserved proteins. To find the topologically similar protein pairs, a similarity measure is necessary for evaluating the topological similarity for each pair of nodes. The mathematical question is how to calculate a similarity of a pair of nodes, which are from two different networks [37]. In the aligner of IsoRank, it was calculated based on the principle that if two nodes are aligned, then their neighbors should be aligned as well. Our method works on a principle that if two nodes are aligned, then the local induced-subgraphs should be similar.

**Fig. 1:**
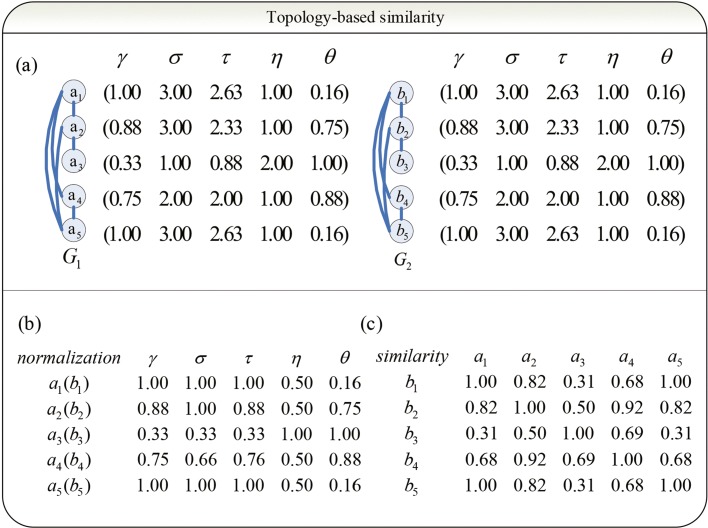
The calculation of similarity matrix between two networks *G*_1_ and *G*_2_. **a** A 5-tuple-feature vector (*γ*,*σ*,*τ*,*η*,*θ*) was calculated on each node. Here, the vector of *γ*, (1,0.88,0.33,0.75,1) ^*T*^, is the normalized major eigenvector of the adjacent matrix of the graph. Vectors of *σ* and *η* are the number of 1-step neighbors and 2-step neighbors for each node. Vectors of *τ* and *θ* describe the influence of each node to their 1-step neighbors and 2-step neighbors. **b** Vectors of *σ*,*τ*,*η*,*θ* were normalized by its maximal element. **c** The similarity matrix was calculated by a Gaussian-based similarity measure $s_{t}(u,v)=exp\left (-\frac {1}{2}x^{2}\right)$. Here, *u* and *v* is a pair of nodes, and *x* is the Euclidean distance between the two feature vectors of *u* and *v*

Given a network *G*=(*V*,*E*),*V*={*v*_1_,*v*_2_,...,*v*_*n*_}, we design a 5-tuple-feature vector (*γ*,*σ*,*τ*,*η*,*θ*) for each node in V to represent local connections of its corresponding node. Without loss of generality, we denote the adjacent matrix of G as M _*n*×*n*_. Since M is real and symmetric, there must exist a major normalized eigenvector K=(k_1_,k_2_...k _*n*_). In another words, K is the normalized eigenvector of the largest eigenvalue. Then, *k*_*i*_,1≤*i*≤*n* represents the reputation of the node v _*i*_. The greater the reputation is, the more important the node is. Therefore, we use k _*i*_ as the first element of the 5-tuple-feature vector (i.e. *γ*) to character the node v _*i*_. Let us denote the neighbor of v as N _*v*_. Then, we use |*N*_*v*_| as the second element of the 5-tuple-feature vector (i.e. *σ*), the sum of the reputation of these nodes $\sum _{x\in N_{v}}k_{x}$ as the third element (i.e. *τ*). Let us denote these nodes that are 2-step away from v as N$_{v}^{2}$. It notes that all nodes in $N_{v}^{2}$ are not directly connected to *v*. Then, we use $|N_{v}^{2} |$ as the fourth element (i.e. *η*). The last element *η* is calculated by the formula $\frac {1}{2}\sum _{x\in N_{v}^{2}}k_{x}p_{xv}$. Here, we denote the number of the shortest paths from x to v as p _*xv*_. As shown in Fig. 1a, there are two networks G_1_ and G_2_. Based on the definition stated above, the 5-tuple-feature vector of *a*_1_,*a*_2_,*a*_3_,*a*_4_,*a*_5_ in *G*_1_ are (1,3,2.63,1,0.16),(0.88,3,2.33,1,0.75),(0.33,1,0.88,2,1),(0.75,2,2,1,0.88),(1,3,2.63,1,0.16), respectively. They are the same for *b*_1_,*b*_2_,*b*_3_,*b*_4_,*b*_5_ in *G*_2_. The vector of each element of all nodes should be normalized in the following step as shown in Fig. 1b. With the normalized 5-tuple-feature vector, the node similarity of any two nodes *s*_*t*_(*u*,*v*) can be calculated with the Gaussian function $s_{t}(u, v)= exp(-\frac {1}{2}x^{2})$, where *x* represents the Euclidean distance between the 5-tuple-feature vector of node u and v. For instance, as shown in Fig. 1a, the vector of *a*_*i*_ and *b*_*i*_ are the same. Therefore, the diagonal of the similarity matrix is (1,1,1,1,1).

### Simulated annealing

To find an optimal network alignment, we applied a linear model to integrate both sequence and topology information. The alignment score can be formulated as $f(\mathbbm {A})=\sum _{m\in \mathbbm {A}}s_{m}$, where $\mathbbm {A}$ and *m* is refer to a global alignment and a matchset, respectively. Suppose *m*={*m*_1_,*m*_2_,...,*m*_*v*_}, the alignment score of the matchset is $s_{m}= \sum _{i=m_{1}}^{m_{v-1}} \sum _{j=i}^{m_{v}} \alpha s_{h}(i,j) + (1-\alpha)s_{t}(i,j)$. By default, *α*=0.5. User can increase *α* when he consider the sequence similarity is more important and decrease *α* when he consider the topological similarity is more important. Therefore, the problem of global network alignment can be modeled as an optimization problem, which is to search for an optimal alignment $\mathbbm {A}^{*}$, such that $\mathbbm {A}^{*}=arg \max \limits _{\mathbbm {A}}f(\mathbbm {A})=\sum _{m\in \mathbbm {A}}s_{m}$.

To solve this problem, we used a simulated annealing algorithm [38] to search for an approximately optimal solution. Simulated annealing is a commonly used approach in the discovering of network alignment solutions, as it can rapidly converge in a favorable time complexity [39]. As shown in the pseudocode of simulated annealing, the alignment A was firstly initialized to an empty set $\varnothing $. Then we repeatedly perturb the current alignment A with a Metropolis scheme P(*Δf*)=$e^{\frac {\Delta f}{(Ti*s)}}$ as the equilibrium distribution till the alignment score converges.


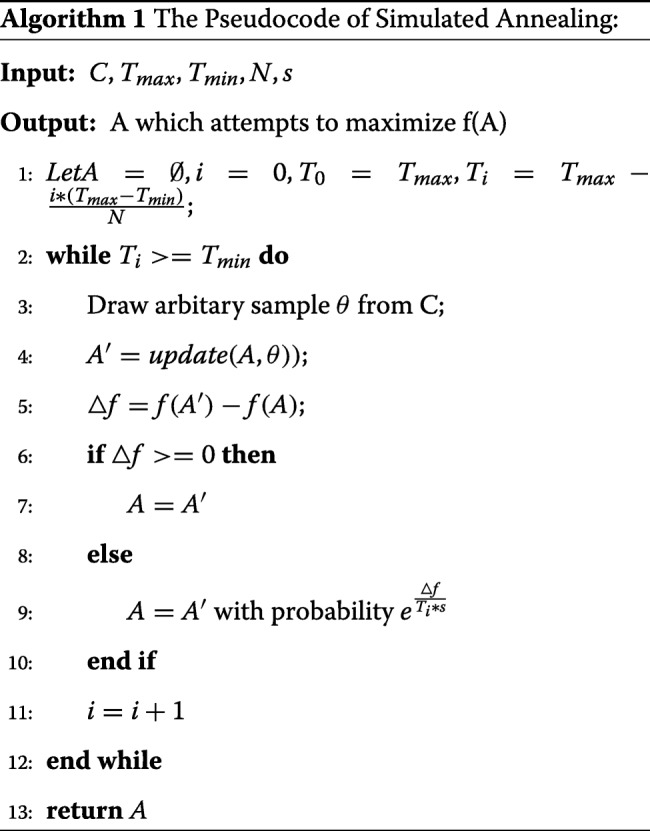


## Result and discussion

### Test datasets and experimental setup

To test our method on real biological data, PPI network of five species were downloaded from the public database IntAct [40] (https://www.ebi.ac.uk/intact/). The five species include mus musculus (MM), saccharomyces cerevisiae (SC), drosophila melanogaster (DM), arabidopsis thaliana (AT) and homo sapiens (HS). Interactions could be detected by different methods, such as ubiquitinase assay, anti tag/bait coimmunoprecipitation. However, some experimental methods such as Tandem Affinity Purification do generate molecular interactions that can involve more than two molecules. An expansion algorithm was applied to transform these n-ary interactions into a set of binary interactions. To improve the data quality, these interactions of the spoke expanded co-complexes are filtered out. As shown in Table 1, 41,043 proteins and 193,576 interactions were collected as test datasets. In order to measure the biological quality for alignment results, we analyzed the functional similarity based on Gene Ontology terms [41], which include molecular function (MF), biological process (BP) and cellular component (CC). The functional annotation data were downloaded from the gene ontology annotation database (GOA) [42]. All of our test datasets can be freely accessible at http://www.nwpu-bioinformatics.com/netcoffee2/dataset.tar.gz.

**Table 1:** Statistics of PPI networks of five species: mus musculus (MM), saccharomyces cerevisiae (SC), drosophila melanogaster (DM), arabidopsis thaliana (AT) and homo sapiens (HS)

Species	NO.nodes	NO.edges	BP Ann.(%)	MF Ann.(%)	CC Ann.(%)
MM	3611	4704	87.03	87.59	88.00
SC	5708	42674	94.55	94.48	90.94
DM	8715	26362	65.81	64.46	64.30
AT	5665	19247	84.99	78.78	78.44
HS	17344	100589	70.14	71.86	72.95

Functional annotations of proteins are collected, which include biological process (BP), molecular function (MF) and cellular component (CC)

We have implemented NetCoffee 2 in C++ using the igraph library (version 0.7.1) [43]. The source code and binary code are freely available on the GitHub repository under the GNU GPL v3 license https://github.com/screamer/NetCoffee2. To compare algorithm performance, we ran our algorithm and three other algorithms NetCoffee, IsoRankN and multiMAGNA++ on a set of real biological datasets. The suggested parameters were used for running all alignment tools. As seen in Table 2, eight datasets were generated as benchmark datasets. The number of PPI networks in eight benchmark datasets ranges from two to five. The biggest PPI network is HS, so we generated datasets based on the follow rules: the datasets include HS or not. dataset1 and dataset2 include two PPI networks, so one dataset includes HS, and another do not include HS. dataset3 to dataset6 include three PPI networks, so two dataset includes HS, and another two do not include HS. To reduce the running time of the algorithm, we generate dataset7 without HS. All the four algorithms were performed on a same machine with CPU Intel Xeon E5-2630v4.

**Table 2:** Algorithms performance were tested on eight datasets, which were represented as D1, D2,..., D8

Species	D1	D2	D3	D4	D5	D6	D7	D8
MM		*√*	*√*		*√*		*√*	*√*
SC	*√*			*√*		*√*	*√*	*√*
DM		*√*	*√*	*√*		*√*	*√*	*√*
AT			*√*	*√*	*√*		*√*	*√*
HS	*√*				*√*	*√*		*√*

### Performance and comparison

Our goal is to identify a set of matchsets that are biologically meaningful. To verify the biological quality of aligment results, we take two aspects into consideration: 1) each matchset is functionally conserved; 2) the alignment node map cover as many proteins as possible. Therefore, we use coverage and consistency to evaluate the biological quality of alignment results. Coverage serves as a proxy for sensitivity, indicating the amount of proteins the alignment can explain. Consistency serves as a proxy for specificity, measuring the functional similarity of proteins in each match set. There is a trade-off between coverage and consistency.

**Fig. 2:**
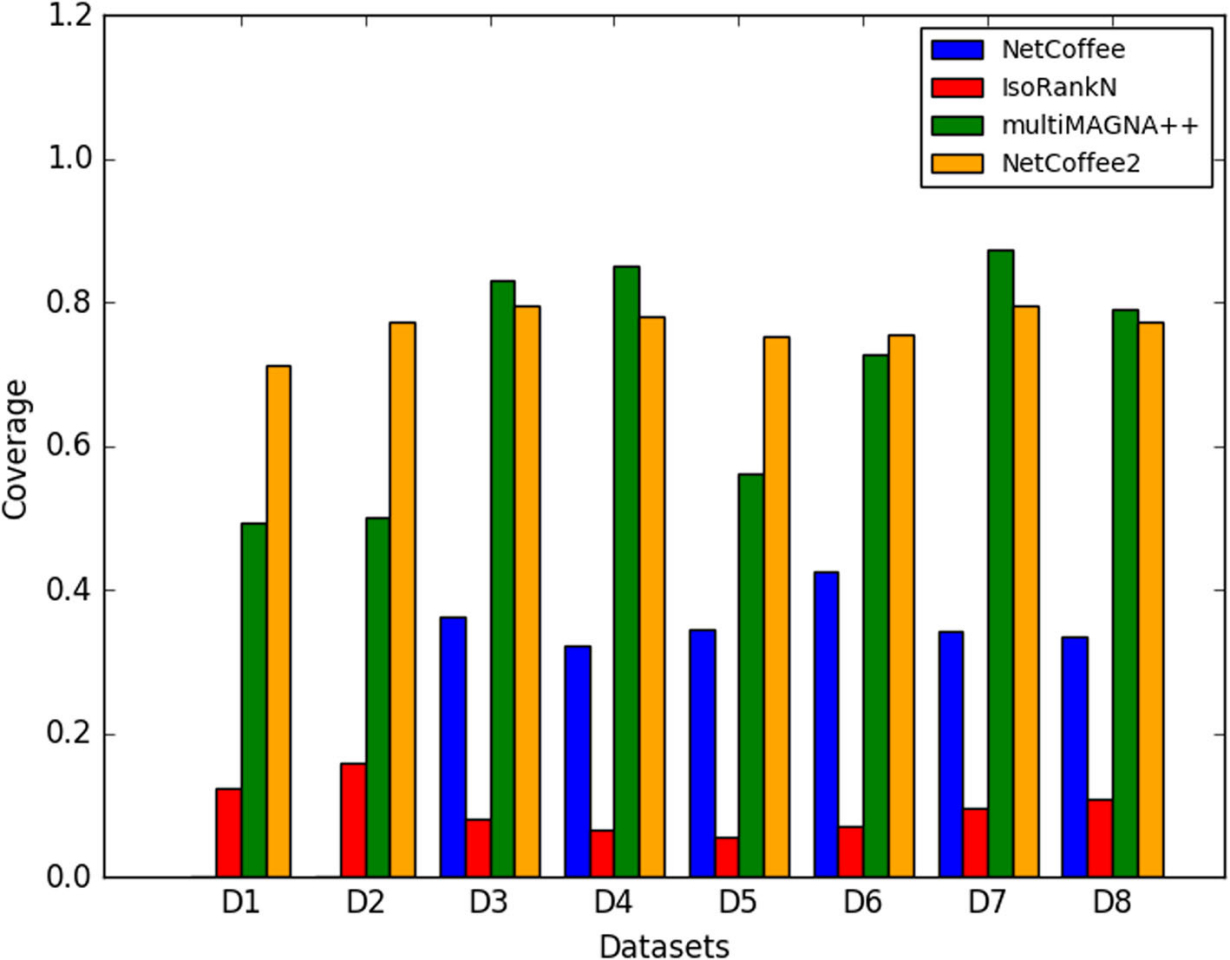
Coverage of NetCoffee, IsoRankN, multiMAGNA++, and NetCoffee2 on eight test datasets. Coverage was measured by the percentage of aligned proteins in alignments

Given an alignment solution, we used the percentage of aligned proteins as coverage. As the number of nodes varies in different networks, some proteins might be lost in a one-to-one node mapping. This can be explained by gene loss events in evolution. And these homogeneous proteins from one species can be accounted for gene duplication in evolution. In our test, multiMAGNA++ is the only algorithm that supports one-to-one node mapping. All other algorithms allow multiple-to-multiple node mapping. As NetCoffee is not applicable on pairwise network alignment, there is no NetCoffee result for D1 and D2. From Fig. 2, we can see that NetCoffee2 stably found a coverage of 76.7% on average for all the eight datasets. It is followed by multiMAGNA++, which found 70.4% proteins on average. Although the coverage of MultiMAGNA++ can be more than 80% on D3, D4 and D7, it rapidly fell to 50% on D1, D2 and D5. NetCoffee approximately identifies about 35% proteins on average, which is less than the coverage of NetCoffee2 and multiMAGNA++. IsoRankN found only an average of 9.6% proteins on eight datasets, which is obviously smaller than the coverage of the other competitor. Overall, the results show that NetCoffee2 is superior to multiMAGNA++, NetCoffee and IsoRankN in terms of coverage and it is more stable than all of its competitors.

Consistency is used to measure the biological quality of matchsets in alignment results. We employed two concepts to evaluate global alignment algorithms based on Gene Ontology (GO) terms: mean entropy (ME) and mean normalized entropy (MNE) [28, 30].Given a matchset *m*={*v*_1_,*v*_2_,...,*v*_*n*_}, the entropy of *m* was calculated by the formula $E(m)=\sum _{i=1}^{d}p_{i}\times log(p_{i})$. Here, *d* represents the number of different GO terms, *p*_*i*_ the proportion of the i^*th*^ GO term in all annotations of *v*. The mean entropy (ME) is the arithmetic mean of entropy for all matchsets. The normalized entropy of *m* is defined as $NE(m)=-\frac {1}{log(d)}\sum _{i=1}^{d}p_{i}\times log(p_{i})$. The mean normalized entropy (MNE) is the arithmetic mean of normalized entropy for all matchsets in a global alignment. It should be noted that these alignments with lower ME and MNE values are more functionally coherent. As can be seen in Table 3, NetCoffee2 has the best performance on D2, D7 and D8 in terms of ME, which are 0.73, 1.01 and 1.10, respectively. And mutliMAGNA++ obtains the best ME on D1 (0.94), D3 (0.91), D5 (0.98) and D6 (1.00). NetCoffee gets the best ME on D4 (0.85) and D6 (1.00). Overall, NetCoffee2 found the best ME (0.973) on average, which is followed by multiMAGNA++ (1.005), NetCoffee (1.022) and IsoRankN (1.144). Furthermore, NetCoffee2 obtains an average of 0.53 in terms of MNE, which is followed by NetCoffee (0.55), multiMAGNA++ (0.56) and IsoRankN (0.58). It outperforms it competitors on all the eight datasets in terms of MNE. Therefore, we can draw a conclusion that NetCoffee2 is superior to the existing algorithms multiMAGNA++, NetCoffee and IsoRankN in terms of both ME and MNE.

**Table 3:** Consistency was measured by mean entropy (ME) and mean normalized entropy (MNE)

Algorithm	Consistency	D1	D2	D3	D4	D5	D6	D7	D8	Average
isoRankN	ME	1.09	1.07	1.15	1.07	1.18	1.20	1.19	1.20	1.144
	MNE	0.58	0.56	0.58	0.59	0.53	0.60	0.60	0.58	0.58
NetCoffee	ME	*	*	0.99	0.85	1.05	1.00	1.07	1.17	1.022
	MNE	*	*	0.54	0.54	0.53	0.55	0.58	0.57	0.55
multiMAGNA++	ME	0.94	0.94	0.91	0.93	0.98	1.00	1.16	1.18	1.005
	MNE	0.55	0.54	0.53	0.58	0.52	0.57	0.63	0.59	0.56
NetCoffee2	ME	1.04	0.73	0.94	0.87	1.04	1.05	1.01	1.10	0.973
	MNE	0.54	0.46	0.52	0.54	0.52	0.55	0.56	0.55	0.53

Notably, a matchset is more functionally coherent when ME and MNE are smaller. There is no result of NetCoffee on D1 and D2, because it can not be applied to pairwise network alignment

## Conclusion

Network alignment is a very important computational framework for understanding molecular function and phylogenetic relationships. However, there are still rooms for improving existing algorithms in terms of coverage and consistency. Here, we developed an efficient algorithm NetCoffee2 based on graph feature vectors to globally align multiple PPI networks. NetCoffee2 is a fast, accurate and scalable program for both pairwise and multiple network alignment problems. It can be applied to detect functionally conserved proteins across different PPI networks. To evaluate the algorithm performance, NetCoffee2 and three existing algorithms have been performed on eight real biological datasets. Gene ontology annotation data were used to test the functional coherence for all alignments. Results show that NetCoffee2 is apparently superior to multiMAGNA++, NetCoffee and IsoRankN in term of both coverage and consistency. It can be concluded that NetCoffee2 is a versatile and efficient computational tool that can be applied to both pairwise and multiple network alignments. Hopefully, its application in the analyses of PPI networks can benefit the research community in the fields of molecular function and evolution.

## Data Availability

Not applicable.

## Abbreviations

GNA: Global network alignment
PPI: Protein-protein interactions
SA: Simulated annealing
